# Cooperative behavior and affect heuristic. When the rationality of love matters

**DOI:** 10.3389/fsoc.2025.1508740

**Published:** 2025-05-13

**Authors:** Albertina Oliverio

**Affiliations:** Division of Social Sciences, Department of Law and Social Sciences, Università degli Studi ‘G. d’Annunzio’, Chieti-Pescara, Italy

**Keywords:** rational choice theory, cooperation, emotions, affect heuristic, cognitive rationality

## Abstract

A central problem of social theory consists in explaining individual cooperative behavior. One of the main interpretations is rooted in the rational choice theory, born from the *homo oeconomicus model*, which proposes instrumental rationality, maximization of expected utility and self-interest as unique presuppositions of individual behavior. These assumptions have been widely criticized, especially concerning their descriptive adequacy in cases of cooperative behavior. Numerous empirical evidence shows how individual behavior can be disinterested in these cases, not based on instrumental calculation and, therefore, explainable on the basis of other dimensions in cognitive human system and individual action. Based on the literature developed in the field of cognitive psychology and experimental economics, the article proposes to analyze cooperative behaviors (conceived in a broad sense since not necessarily implying shared goals), considering components of the rationality of a cognitive and emotional nature, using the concept of *affect heuristic*. It shows how some emotional behavioral responses (e.g., love, fairness moods, etc.) can be crucial in explaining individual selfless behaviors and their role in developing cooperation. Our analysis is developed in the light of a broader cognitive system and a more extended notion of rationality than the strictly economic one.

## 1 Introduction

December 1938, only a few days before Christmas. British stockbroker Nicholas Winton is about to leave for Switzerland on holiday, but is contacted by a friend who is in Prague on behalf of the British Committee for Refugees from Czechoslovakia who asks to join him to help with the ongoing humanitarian crisis. Upset at the plight of so many Jewish families, Winton decides, at his peril, to cooperate in a plan designed to help those wishing to send their children to Britain. Amid Nazi expansionism, he tackles the complex British bureaucratic machine to find families to take in the children. Through his efforts and courage, along with those of many others involved in this project, Winton saves the lives of 669 children. Nicholas Winton’s story is a very touching one, the manifestation of what common sense would let us call ‘selfless love’ for others, which would seem to be confirmed by the fact that Winton never spoke about these events until much later in 1988, when his wife found notes with the names of all the children he had rescued. History and everyday life teach us that this type of behavior is by no means unique: taking World War II as an example, we could cite many other episodes in which certain individuals, often at the risk of their own lives, acted according to what could be broadly defined as a ‘cooperative’ spirit toward other human beings and, more generally, toward society and life, demonstrating sensitivity, empathy, altruism, solidarity, in short, love. Love behavior therefore seems to translate into a form of social action with specific characteristics, as sociological literature has long pointed out (Sorokin, 1954; Boltanski, 1990), and recently recalled (Araújo et al., 2016; Cataldi and Iorio, 2023; Cataldi et al., 2024; Galindo Filho, 2011; Iorio, 2014), not remaining simply a feeling. These kinds of theoretical reflections constitute the starting point from which this article develops, namely the hypothesis that many cooperative behaviors cannot be reduced to an analysis in terms of economic rationality, and that they should be explained in a broader sense: not only behaviors oriented to shared goals among individuals, but also actions in which this sharing is lacking, but motivated by a kind of cooperative spirit toward the wellbeing of others resulting in altruism, generosity, and love. We therefore assume that cooperative behaviors can be read and explained in the light of a broader view of cognitive rationality that integrates the different components of action, including the affective one (Boudon, 1995). Consequently, this paper aims to reflect on the type of logic underlying a specific kind of social action of love, the one translating in cooperative behaviors, in relation to the multiple debate on rational choice theory’s economic rationality notion (paragraph 1), on the generality of the concept of self-interest through examples of behaviors that common sense does not allow us to attribute to economic calculation (paragraph 2), on the importance of the affective component in explaining cooperative behavior in the light of a broader view of cognitive rationality on the basis of which emotional behavior cannot be reduced to simple feelings (paragraph 3).

## 2 Cooperative behavior and *homo oeconomicus*

A central problem of social theory consists in explaining individual cooperative behavior, which brings us back to that of Mr. Winton. One of the main interpretations is rooted in the ‘Rational Choice Theory’ (RCT), born from the ‘homo oeconomicus model’, which proposes instrumental rationality, maximization of expected utility theory, and self-interest as unique presuppositions of individual behavior. The *homo oeconomicus* model is a dominant model that originally developed in the field of economics and rests on a notion of ‘economic’ or ‘instrumental’ rationality. This meaning of rationality, which does not imply any ethical content, indicates what criteria an individual should follow to act, precisely, rationally and is formally described with logical-mathematical models. To simplify, we could say that according to it, the individual has perfect information about the alternatives of choice and their consequences, as well as an adequate cognitive capacity to evaluate the different options by comparing them with each other, to identify a relationship that links them to their respective consequences, to order these consequences from the best to the worst, and to select the best action (means) on the basis of a rational calculation aimed at maximising the ‘advantages’ or ‘benefits’ and minimising the ‘disadvantages’ or ‘costs’. The logic of the *homo oeconomicus* model has been successful both in the study of individual behavior in certain contexts and, above all, in the analysis of situations characterized by risk and uncertainty. The first to proceed in the latter direction were John von Neumann and Oskar Von Neumann and Morgenstern (1944) who developed a theory of rational choice under conditions of risk known as the ‘Expected Utility Theory’ (EUT). The EUT is based on a series of logical consistency axioms that characterize the rationality of an individual’s actions (stable and coherent preferences), and on the principles of the calculation of probabilities. In this sense, the individual who acts is represented as a ‘Bayesian actor’ capable of assigning probability values to events. It is from these axioms that Von Neumann and Morgenstern mathematically demonstrated the existence of an expected utility function given by the sum of the utilities associated with possible outcomes multiplied by their probabilities of occurrence. Individual maximization of expected utility (i.e., maximization of one’s own utility function) is a rational criterion for choosing between risky alternatives. According to a current representation in the social sciences and sociology in particular, a very similar reformulation of the EUT is RCT. As we said, RCT is the dominant framework in the social sciences for explaining the rise of cooperative behavior. As a matter of fact, some social scientists have attempted to propose RCT and the hypotheses of cost/benefit analysis and utility maximization as a general interpretative model of individual behavior (Becker, 1996; Coleman, 1990). EUT and RCT were dominant models until the last decades of the last century as they have been recognized by many social scientists as effective theories in accounting for human behavior in numerous situations insofar as they provide a clear, concise and simple vision of the human nature they presuppose. Nevertheless, they have aroused numerous perplexities and criticisms, generating a wide debate both with regard to the notion of instrumental rationality and its presuppositions, and, more generally, with regard to the theme of the explanation and description of human behavior. These perplexities and criticisms involve precisely the problem of the explanation of particular types of behavior that common sense would let us define as ‘disinterested’, such as those of a cooperative, altruistic or solidaristic nature, or that refer, in a general sense, to love. In the sociological sphere, for example, important reflections have focused on outlining a series of indispensable postulates on which RCT rests and on highlighting its explanatory and descriptive ‘restrictiveness’ (Boudon, 1996 and 2003). These are the consequentialist one (according to which the meaning of action for the actor always resides in the consequences of his actions), the egoist one (according to which among the consequences of individual action, the only ones that concern the actor are those that affect him personally), and the cost–benefit one according to which individuals are invited by the context to act according to their own ‘interest’ with a view to maximizing it on the basis of cost–benefit analysis. These postulates, while making RCT indispensable when it comes to explaining a large number of phenomena, would not allow it to have the status of a general theory and would render it impotent in understanding and explaining many others phenomena, including those that bring into play individual beliefs and behaviors such that it is just against common sense to suppose that they could be dictated by a selfish attitude. Referring back to Max Weber’s vision of understanding sociology, these considerations are based on the conviction that understanding the reasons for beliefs and actions is an essential moment in social explanation: it is therefore necessary for this to take place within a broader theoretical paradigm that only in some cases can be traced back to the three postulates of RCT making its axiomatics relevant (Boudon, 2003), but in many other cases must widen the mesh of the postulates on which RCT rests. As far as the assumptions underlying the EUT’s view of rationality are concerned, they have been questioned first and foremost by economists themselves with regard to their descriptive capacity for actual individual behavior (Allais, 1953; Ellsberg, 1961). Soon, moreover, research in cognitive psychology and experimental economics revealed systematic violations of the axioms of the EUT (Kahneman and Tversky, 1979; Kahneman et al., 1982). On the basis of this research, new models for explaining individual action have been advanced that are endowed with greater descriptive capacity and are often centered on different notions of rationality. Some of these notions are based on the hypothesis that individuals act on the basis of a ‘limited’ rationality with respect to economic rationality (Simon, 1982) as far as knowledge and cognitive capacities are concerned; others, as mentioned, are aimed at defining a more ‘extended’ concept of rationality that, in addition to self-interest, also takes into account the axiological and cognitive reasons that actors have for behaving in a certain way (‘axiological rationality’ and ‘cognitive rationality’: Boudon, 1995).

## 3 Beyond the self-interest

An extensive literature in the social sciences, and in sociology in particular, argues that the concept of self-interest, made famous by Adam Smith’s *The Wealth of Nations* (1776) and central to the classical conception of rational behavior, cannot therefore claim generality in the explanation of human action (see for example: Boudon, 1995; Elster, 2007; Rabin, 1998). The pursuit of self-interest is only one possible motivation that drives individuals to act: it is probably a recurring motivation that allows many situations and actions to be read in terms of economic rationality, but nevertheless it is not the only one. Individuals may be motivated by reasons other than utilitarian and instrumental ones, such as those based on different beliefs, values and moral principles that do not seem reducible to economic logic. An example of this is the so-called ‘voting paradox’. According to this paradox, if one were to adhere strictly to the postulates on which RCT rests and, in particular, to the central one of consequentialism, one would not understand why people vote: it would be a very costly action (wasting time to go and vote instead of devoting oneself to more useful or more interesting activities) for very little benefit since the individual vote has but a practically nil chance of influencing the outcome of a popular consultation (Boudon, 1997). However, despite these considerations, people vote. It is a paradox that has become the stumbling block par excellence on which RCT slams, and which has thus given rise to a vast literature and multiple attempts at solutions (see for example: Ferejohn and Fiorina, 1974; Overbye, 1995). Explaining voting behavior outside an instrumental and utilitarian view of rationality does not mean surrendering to irrational causes, but may mean relying on a more general theory of rationality that refers to an axiological component of action along the lines of Max Weber (2013) famous definition of *Wertrationalität*. In practice, there can be very strong reasons to believe in the goodness, rightness, legitimacy, etc., of a set of principles or norms. And this allows us to understand how in some circumstances individuals act on the basis of reasons that go beyond the mere concept of self-interest: that is, that in some cases action is guided by principles rather than by the consequences it is likely to entail (Boudon, 1997). Another example of what we are talking about is ocity behavior. It may indeed be interesting to consider the case of those situations in which individuals act cooperatively on the basis of reciprocity even when this is against their own self-interest. In a series of experiments (Fehr and Gächter, 2000a), it has been shown, for example, that individuals who receive generous treatment even from strangers still respond to it generously even if they cannot expect any gain or advantage or if it is costly for them. Similar experiments have also established a systematic tendency for individuals to repay rude or hostile behavior with similar behavior, even if it does not benefit them (Fehr and Gächter, 2000b). It is reciprocity-based behavior that constitutes a falsification of the self-interest hypothesis conceived as the sole motive behind behavior within the homo economicus model as it is not motivated by future gains or benefits. The results of such experiments have revealed how many behaviors are due to reciprocity and not calculation; how cooperative habits can persist through reciprocity; how groups in which such habits have developed can be more economically successful than those in which more selfish behavior is the norm. An example of a situation that helps us understand ‘negative’ reciprocity behavior is the so-called ‘ultimatum game’. In it, an asset, e.g., a sum of money, is to be divided between two individuals. One of the two (proposer), must give the ultimatum, i.e., make a proposal to the other (responder). The latter can only accept or reject the proposal: if he accepts it, the proposer gets what he asks for and the responder what remains; if he rejects it, neither of them gets anything. The solution that generally emerges from this game consists of an equal division of the sum played. This solution, instead of reflecting the strict logic of utility maximization according to which the proposer should propose unequal subdivisions to its advantage since the responder would in any case have an interest in accepting them, may for instance be justified by beliefs, reasons, values and social conventions referring to the principles of fairness, loyalty or reciprocity. It should be added that several experiments have shown that proposers who offer the responder less than 30% of the sum are rejected with a very high probability (Camerer and Thaler, 1995; Roth, 1995; Gintis et al., 2005), and the empirical evidence shows that this happens regardless of the countries or geographical areas in which the experiments were carried out (Slonim and Roth, 1998; Cameron, 1999; Henrich et al., 2001). Most individuals therefore seem to approach the ultimatum game on the basis of ethical principles both in making fair proposals and in punishing offers considered unfair. These are clearly behaviors that do not tend to maximize the expected monetary gain and constitute a violation of RCT (a very interesting perspective on non-contractual reciprocity is developed in: Bruni, 2006). An interdisciplinary look at the topic leads us to highlight how the above empirical evidence also seems to find confirmation in the field of neuroscience. Brain magnetic resonance imaging of individuals taking part in the ultimatum game highlighted how the most ‘unfair’ offers mainly stimulate the frontal insula responsible for negative emotions such as discomfort and disgust (Rilling et al., 2002; Sanfey et al., 2003). High stimulation of these areas can therefore be held ‘responsible’ for a rejection of an unfair offer due to the prevalence of an ‘innate’ sense of justice.

The topic of reciprocity is also closely related to that of ‘gift’ around which a large literature and lively debate has developed, and which constitutes another valid example of behavior that many consider not reducible to self-interest maximization and instrumental rationality. Classical studies in this field, as is well known, have highlighted how in some archaic societies (Polynesians, Melanesians, some Indian tribes in Canada, etc.) gift-giving behavior is central to a system of ceremonial exchange and rests on an obligation of reciprocity involving honor and prestige whose symbolic function creates social bonds (Mauss, 1923–1924). It is a system of exchange, that of the gift, that constitutes a total social fact in the sense that it involves all spheres of society in terms of power relations, sociability, rivalry, trust, etc. Positions in tune with this explanation of gift behavior are shared by several authors who criticize the idea that the gift is economically concerned and obeys the same logic as utilitarian exchanges (Caillé, 2000; Godbout, 2000; Godelier, 1996). The examples of the voting paradox, reciprocity behavior and gift stimulate reflection, from different points of view, on other explanatory components of behavior that go beyond self-interest: good reasons, values, social and moral obligations, but also, for example, emotions. In the sociological sphere, an important perspective is the one that refers to the more general theme of the moral dimension of action, according to which individual behavior would also be characterized by a moral dimension, value commitments, and affective-emotional involvements concerning a social and community level with the role of stimulating and encouraging moral obligations (Etzioni, 1988). The normative-affective (non-cognitive) factors would reflect individual and collective (community) processes that significantly govern cognition, reasoning, and evaluation that precede individual action.

## 4 Heuristics and affect as a different possible explanatory scenario

An even different perspective that goes beyond self-interest and aims to reconcile emotions and the cognitive component comes from cognitive psychology and experimental economics. In particular, in this field numerous empirical evidence shows how individual behavior can be disinterested, not based on instrumental calculation and, therefore, explainable on the basis of other dimensions in cognitive human systems and individual action. Behavior inconsistent with the logic of interest is explained on the basis of the hypothesis that information processing by individuals can often be influenced by cognitive limitations, simplifying strategies and emotional factors. Therefore, it is hypothesized that individuals resort to cognitive heuristics when evaluating and acting (Tversky and Kahneman, 1974; Kahneman et al., 1982). These are rules-of-thumb, strategies that individuals rely on more or less consciously in the simple and rapid resolution of a variety of tasks and problems in order to make best use of their limited cognitive capacities (this aspect necessarily also implies the study of the ecological rationality of heuristics in order to explain their usefulness, see Gigerenzer and Gaissmaier, 2011). The prerogative of heuristics is the so-called ‘system 1’ from which quick and often summary evaluations result (Kahneman, 2011). In contrast, ‘system 2’ is based on reflection, effort and is more precise. Although heuristics are regarded as useful means to ‘economise’ the limited capacities of individuals and produce evaluations that are correct and work in a large number of situations, they can sometimes lead to systematic errors of evaluation, the cognitive biases, that are recurrent, persistent and sometimes followed by bad consequences. This has been highlighted, for example, by the empirical evidence concerning the three main heuristics/biases: the availability heuristic (the tendency to consider the easiest events to remember as having a very high frequency or probability), the representativeness heuristic (the tendency to rely on stereotypes in evaluations and predictions) and the anchoring heuristic (the tendency to use an arbitrary starting point as a constant reference in the evaluation process). The biases arising from the use of these heuristics are normally due to the violation of basic statistical principles and the incomplete or incorrect treatment of available data. For example, in a classical study on risk perception (Lichtenstein et al., 1978) a sample of individuals were asked first to estimate the frequency of different causes of death (illness, tornadoes, car accidents, etc.), second to estimate the frequency of these causes with respect to a specific cause whose objective frequency was given, and finally to estimate between two causes of death which was the most frequent. The results obtained showed that individuals were making serious biases due to the availability heuristic. Resorting to the heuristic in question, individuals were in fact judging the frequency of each type of death based on events and circumstances they remembered best. However, in most of the answers given, there was no correspondence between stated subjective frequencies and objective frequencies. Rare but dramatic and sensational causes of death (accidents, homicides, etc.) were overestimated especially when they caused a large number of victims at once (natural disasters, etc.), while more common and unspectacular causes, especially those that involved one death at a time such as diseases (diabetes, emphysema, heart attack, etc.), were underestimated.

Understanding these cognitive strategies also helps to describe and explain behavior outside the logic of the postulates ascribable to cost/benefit analysis and utility maximization and to identify components and reasons for the rationality of action of a different nature. Returning to the analysis of cooperative behavior, it is, for example, plausible to hypothesize reading them in the light of the so-called affect heuristic. The latter is a form of evaluation that relies on an ‘automatic’ and fast cognitive system (system 1) on the basis of emotions (affect). Experimental results have shown that when faced with a rapid assessment of a certain situation, of the risks and benefits of a given behavior, it is often our affective states, i.e., our emotions, that can determine it, resulting in a choice (Finucane et al., 2000; Loewenstein et al., 2001; Slovic et al., 2007; Slovic et al., 2002). Research has shown that this occurs both in the case of incidental affect (the affect that is not connected to an evaluation or an action, but depends on external circumstances such as the meteorological ones), and in the case of integral affect (the affect that is part of the internal representation of the situation by the perceiver and allows for differentiation between good and bad options), and, finally, in the case of a combination of the two (Loewenstein and Lerner, 2002; Schwarz, 2001; Västfjäll et al., 2016). This heuristic can manifest itself in situations of urgency, stress, fatigue, lack of motivation and result in bad decisions. But in reality, it often also produces very positive outcomes, and we think it is plausible to assume that it can stimulate cooperative behavior. In both economics and sociology, theories of rational action (EUT and RCT) have in common that they regard emotions as obstacles to action and life in society as a whole. However, the importance of emotions in the transition to action has been debated for several decades: it has been argued that affective reactions are often the first reactions to stimuli on the basis of which information processing and evaluation subsequently take place (Zajonc, 1980). Emotions can influence our behavior by motivating action and anticipating the emotional responses of others. In the case of cooperative behavior, this ‘emotional shortcut’ may take the form of an action that reciprocates certain benefits or costs, that renounces defection from cooperation, or that privileges an unconditional love-component that often takes a back seat to slower and more complex evaluative processes. This is a love-component that, although not treated as a ‘social force’ with a public dimension in the sense of the term ‘social love’ (Cataldi and Iorio, 2023), nevertheless describes a situation in which subjects ‘exceed’ in giving unconditionally with respect to the situation they find themselves in without any corresponding benefits. In the context of this conceptual analysis, it should also be recalled how the theoretical perspective of social love just mentioned promotes new developments in the explanation of action in the absence of reciprocity ascribable to a public dimension of emotion rather than the strictly private one of affect heuristic here discussed. The four dimensions of social love (overabundance, care, universalism, recognition of the other) find support within an empirical literature that also proposes a World Love Index aimed at the operationalization of individual and collective overabounding loving attitude (Palmieri and Iannaccone, 2023).

In essence, going back to the concept of affect heuristic helps showing how some emotional behavioral responses (e.g., love, fairness moods, etc.) can be crucial in explaining individual selfless cooperative behavior. Affect heuristic is thus a cognitive shortcut that, on the basis of some emotional states, can ‘stimulate’ a wide range of cooperative behaviors as an essential component in many forms of evaluation and action. In practice, in the course of the evaluative process individuals refer to an ‘affective pool’ that contains all the positive and negative aspects associated with perceptions and this, especially in some circumstances, may be easier, quicker and more efficient than a process that involves weighing pros and cons or trying to retrieve relevant elements from memory or reviewing all available information (Finucane et al., 2000). This is not to deny the role that controlled cognitive processes of the system 2 type can play in behavior. It is to recognize the importance of both cognitive systems, the combination of which gives rise to a multitude of complex behaviors. One system mediates the other (system 2 not only ensures more analytical reasoning, but at the same time has a control function over system 1 where too much impulsiveness might hide negative automatisms) and this mediation is at the basis of a social life in which cooperation, as we partly mentioned earlier, takes on articulate and mostly disinterested forms. Reasoning about the emotional component of cooperative behavior with reference to an intuitive and coarse system 1 also means dealing with the issue of the origin of this intuitive system, of the heuristics that arise from it such as that of affect, of the behaviors into which it translates such as cooperative ones. We can just touch on the subject of this last point, although it is not the purpose of this essay to delve into it here. One explanation is the biological one. Certainly, even very complex forms of cooperation exist in nature and are to be explained from a biological perspective, i.e., the advantage they present in an evolutionary sense. One need only think, for example, of forms of cooperation between genetically related individuals (theory of parental selection, Hamilton, 1964 and Dawkins, 1976), or between members of different animal species who, although not related, cooperate on very specific tasks aimed at the survival of their species (reciprocal altruism, Trivers, 1971). In nature there are even forms of cooperation between members of different species, but most forms of cooperation in the animal world are qualitatively very different from human behavior. Only humans, in fact, cooperate on complex projects or in ways that invoke an axiological and cognitive component (reasons, principles, values, feelings, etc.). In the human world, the biological, cognitive, and socio-cultural components interact with each other in ways that should not be underestimated. Consider for example, again with reference to cooperative behaviors, the experimental results that highlight the influence that certain social circumstances can have on them. A classic study (Milgram, 1970), for example, showed that in large urban contexts the individual rate of cooperation (e.g., through help/support/solidarity behavior) was negatively correlated with the rate of urban density. In addition, education and learning are central to the explanation of cooperative behaviors. Empathy itself, which is considered a central factor in the explanation of these behaviors, is according to many not just the result of genetic programming, or mere instinctive reactions, but can be learned.

## 5 Conclusion

What has just been said leads us, in conclusion, to reflect further on the concept of rationality that has already been recalled several times in this essay: assigning affect a possible role in explaining certain types of behavior (e.g., cooperative behavior conceived in a broad sense) at the expense of the logic of self-interest means that a sort of ‘rationality of love’ matters. When on the basis of an affective reaction something is ‘felt’ to be pleasurable, right or good, evaluations and behavior in tune with these emotions may ensue, even in those cases in which the action that follows may be costly or contrary to the self-interest. This may help us to more effectively describe and explain cooperative selfless behavior toward others that puts one’s own life at risk (as in the case of Mr. Winton’s selfless love behavior remembered at the beginning of this text), or that results in a manifestation of reciprocity where this may not involve maximizing one’s own utility, or in a gift action where nothing is expected in return. As already mentioned in the introduction, in the sociological explanation it is important to give space to disinterested love behavior conceived as a form of social action with specific characteristics (Sorokin, 1954; Boltanski, 1990; Cataldi and Iorio, 2023). And, albeit with a theoretical approach that is more centered on the mechanisms underlying action, it is precisely from this observation that this paper aimed at two goals. Forst, reviewing a partial body of empirical evidence and literature that allowed us to check the hypothesis according to which many cooperative behaviors cannot be reduced to a single analysis in terms of economic rationality. And second, to highlight how a broader view of rationality integrating different components of action including the affective one is fundamental in order not to limit cooperative behavior to either simple economic calculation or simple feelings. It is evident that only in some situations does affection tout court constitute the stimulus to action. Empirical evidence shows, in fact, as mentioned above, that system 2 plays an important role in our actions, although this does not necessarily limit them to economic behavior. In fact, there is a cognitive rationality broader than the economic one that can also account for actions motivated by strong beliefs, values and ethical principles that are often closely linked to the theme of cooperation, altruism, solidarity and reciprocity. Even in those cases in which it is affection that stimulates action, the emotional component need not necessarily be considered as an anonymous psychic force, but rather can be regarded as one of the components of a broader cognitive system that belongs to a vision of rationality that has already been referred to above, the cognitive one. Often, in fact, feelings and emotions are associated with reasons stemming from principles that can easily be considered as acceptable and that explain how something can be perceived as good, legitimate, just, etc. (Boudon, 1995). Behavior such as selfless cooperation can thus be motivated by a strong affective dimension, but at the same time be founded on socially and culturally rooted systems of reasons, beliefs, principles, and the affective reaction may be all the stronger the more solid the reasons appear to the individual.
